# Payments and quality of care in private for-profit and public hospitals in Greece

**DOI:** 10.1186/1472-6963-11-234

**Published:** 2011-09-23

**Authors:** Elias Kondilis, Magda Gavana, Stathis Giannakopoulos, Emmanouil Smyrnakis, Nikolaos Dombros, Alexis Benos

**Affiliations:** 1 Greek Observatory on the Privatization of Health Care, Aristotle University, 54124 Thessaloniki, Greece; 2 Laboratory of Hygiene, Medical School, Aristotle University, 54124 Thessaloniki, Greece; 3 Medical School, Aristotle University, 54124 Thessaloniki, Greece

## Abstract

**Background:**

Empirical evidence on how ownership type affects the quality and cost of medical care is growing, and debate on these topics is ongoing. Despite the fact that the private sector is a major provider of hospital services in Greece, little comparative information on private versus public sector hospitals is available. The aim of the present study was to describe and compare the operation and performance of private for-profit (PFP) and public hospitals in Greece, focusing on differences in nurse staffing rates, average lengths of stay (ALoS), and Social Health Insurance (SHI) payments for hospital care per patient discharged.

**Methods:**

Five different datasets were prepared and analyzed, two of which were derived from information provided by the National Statistical Service (NSS) of Greece and the other three from data held by the three largest SHI schemes in the country. All data referred to the 3-year period from 2001 to 2003.

**Results:**

PFP hospitals in Greece are smaller than public hospitals, with lower patient occupancy, and have lower staffing rates of all types of nurses and highly qualified nurses compared with public hospitals. Calculation of ALoS using NSS data yielded mixed results, whereas calculations of ALoS and SHI payments using SHI data gave results clearly favoring the public hospital sector in terms of cost-efficiency; in all years examined, over all specialties and all SHI schemes included in our study, unweighted ALoS and SHI payments for hospital care per discharge were higher for PFP facilities.

**Conclusions:**

In a mixed healthcare system, such as that in Greece, significant performance differences were observed between PFP and public hospitals. Close monitoring of healthcare provision by hospital ownership type will be essential to permit evidence-based decisions on the future of the public/private mix in terms of healthcare provision.

## Background

### Debate on how hospital ownership type influences the quality and cost of healthcare

Debate on the effects of hospital ownership type on the quality and costs of medical care has continued for years [1,2]. Pro-private-care advocates argue that involvement of the private sector in healthcare provision affords a fast and innovative response [3], aggressive and creative fulfillment of customer demands [4,5], and a high quality of care [3-6] at competitive prices [4], whereas public health services are inequitable and inefficient towing to bureaucracy, politicization, lack of health worker incentives [7], and resource limitations [1], especially in times of fiscal austerity [8].

Conversely, some claim that, in modern society, some aspects of life are off-limits to commerce and, of these, "healthcare is too precious, intimate and corruptible to entrust to the market" [9]. Such critics voice several arguments and concerns. They claim that for-profit organizations have a strong incentive to engage in opportunistic behavior, especially in a context of informational asymmetry [10]. For example, lack of knowledge renders patients unable to assess the technical quality of services [11,12], making it possible for profit-maximizing companies to misrepresent and undersupply hard-to-monitor aspects of quality of care [2,13,14], to increase profits. Additionally, it has been argued that for-profit facilities tend to offer services at higher prices and to receive higher payments either owing to exploitation and induced demand [15-17] or due to high administrative costs that reflect enormous executive incomes and meticulous attention to financial profit [9,18]. On the other hand, public nonprofit facilities are perceived as offering low-price services because such facilities enjoy tax exemptions [19] and have significantly lower administrative costs [20]. At the same time, because profit is not the key motive, quality assurance and patient safety are thought to be assured [2,21].

Against such a background, some authors have concluded that economic theory is ambiguous and does not clearly show a relationship between ownership and quality of care [22]. Others highlight that, as the pressures on nonprofit and for-profit facilities increase in an environment of enhanced competition, any variation in cost-efficiency (for example, in terms of price [23] or the cost of uncompensated care [24]) between public and private facilities becomes significantly reduced or even reversed.

### Evaluation of health care providers by ownership type: International evidence

Comparative quality evaluation is a common research practice especially in countries such as the USA where the private sector plays an important role in the healthcare system [25,26]. Studies from the USA have sought to compare the performance of nonprofit and for-profit healthcare providers. In most instances, the performance of nonprofit facilities (general and psychiatric hospitals, dialysis units, and nursing homes) was better in terms of mortality rate, health outcomes, cost, and charitable care than was that of their for-profit counterparts [25-31].

In Europe, relevant evidence on the comparative performance of public and for-profit health providers is limited and inconclusive. For example, in the UK, evidence shows that Independent Sector Treatment Centers provide poor-quality data [32,33] and cherry-pick low-risk patients [33-35], compared with NHS hospitals. In Germany, private for-profit (PFP) hospitals are less efficient than public institutions [36,37]. This may be related either to the lack of teaching activities (thus affording less clinical experience to staff [36]) or to the higher average length of stay, probably attributable to patient exploitation [37]. Data from Spain and Italy reveal that cesarean section rates are significantly higher in private clinics compared with public hospitals [38-41] because the financial imperative of the private sector directly or indirectly influences clinical decisions [39]. Additionally, empirical evidence from inpatient psychiatric facilities in Italy shows that private providers offer a lower equivalent full-time staffing level per bed [42] and have a higher average length of patient stay [42,43]. These features reflect facility ownership status rather than patient symptom severity [43]. Recent evidence from nursing homes in Italy and Sweden shows that private elderly care providers are more efficient [44], are associated with less employees per resident [45] and seem to emphasize service aspects rather than structural prerequisites for good care [45] compared to public facilities. Finally, in Catalonia, no statistically significant difference in surgical mortality was observed between public and private open-heart surgery centers [46]. Although such evidence is generally in line with results from the US healthcare system, the data are few, surprisingly indicating that, in most European countries, comparative performance evaluation is not yet well developed as a research and policy practice.

### Evaluating public and PFP healthcare providers in Greece: The evidence to date

Despite the fact that the private sector is a major provider of outpatient and hospital services in Greece, little is known about the performance of this sector compared with that of public facilities. The rate for example of cesarean sections is 15-27% higher, depending on the study, in private than in public maternity hospitals [47,48]. Responsiveness of the private health sector is better than that of the public sector in terms of waiting time, waiting lists, and patient accommodation [49]. Finally, a recent study comparing PFP and public dialysis units in Greece found that the former were overall more efficient than the latter [50].

The aim of the present study was to describe and compare the operation and performance of PFP and public hospitals in Greece, focusing on differences between nurse staffing rates, average lengths of stay (ALoS), and SHI payment per discharge. Such differences can provide useful information on the current performance of hospital service providers by ownership type and thus assist health policy makers to select the appropriate public/private mixture, and the future roles for the two sectors in a mixed healthcare system, such as that of Greece.

## Methods

### Data collection

To describe and compare, on a national level, the operation and performance of PFP and public hospitals, five different datasets were used. Two datasets contained information derived from the National Statistical Service (NSS) of Greece and three gathered data from the three largest SHI funds in the country. All datasets cover the 3-year period from 2001 to 2003.

The NSS data were collected using two surveys (the Annual Hospital Survey and the Annual Patients Discharged Survey) conducted on a yearly basis by the NSS Section of Health, Welfare, and Social Insurance Statistics. Both surveys employ questionnaires completed on a mandatory basis by all hospital providers in the country. The results are made public, separately, with delays of 2 and 4 years, respectively. Until recently, data from both surveys were only partially available after a delay of 6 years. The most recent NSS health and social welfare survey data appeared in 2004; the 1998 data were included [51]. The NSS information contains: (a) the numbers of hospitals, hospital beds, and hospital departments evaluated; (b) the numbers of patients discharged by hospital ownership type, capacity, specialty, and location; and, (c) the numbers of medical, nursing, and auxiliary staff by hospital specialty and location [51]. For the purposes of our study, NSS, following our written requests, provided all data from the unpublished 2001-2003 surveys (the Annual Hospital and Annual Patients Discharged Surveys for the 3 years) as well as additional not-intended-for-publication data (gathered in the same surveys) on numbers of nursing staff and days of hospitalization by hospital ownership type, specialty, and location. The data contained only aggregate information by hospital ownership type, specialty, and location. No hospital- or patient-level information was available.

The SHIs of Greece provide mandatory healthcare coverage, organized on an occupational basis. In 2003, 35 different SHI funds were operating in the country, financed via employer and employee contributions and state subsidies [52]. In some instances, SHI funds run their own primary care and hospital facilities, but, in most cases, services to beneficiaries are provided via contractual arrangements with public and private healthcare providers. In return, SHI funds compensate contracted primary care providers on a fixed (by the state) fee-for-service basis and hospital providers on a fixed per diem basis, plus additional fee-for-service payments (excluded from the per diem fee) for specific diagnostic and therapeutic procedures (such as CT and MRI scans) performed during hospitalization.

No official SHI information system, capable of generating comprehensive data on resource flows and the performance of either public or private contracted providers, exists. All SHI schemes produce (annually) only provisional, aggregated data on beneficiary numbers, and expected revenue and expenditure for the forthcoming fiscal year [52]. Additionally, some funds separately publish balance sheets, with delays of 2-5 years [53,54], depending on the capacity of each fund to monitor contracted providers and expenses and to collect and process the relevant data. No disaggregated information on outgoings or payments is given; it is thus not possible to identify the ownership type of contracted providers. In other words, each SHI fund uses individualized policy and methodology for collection of data, processes the information very slowly, and does not explore potential efficiency differences between public and private contractors.

For the purposes of the present study, were performed a 3-year survey (from June 2005 to June 2008) involving the three largest SHI schemes in Greece. These are (a) the Institution of Social Security (IKA) which insures urban blue- and white-collar workers and their dependents; (b) the Organization of Agricultural Insurance (OGA) which insures farmers and others living in rural areas; and, (c) the Greek Professionals and Tradesmen Fund (TEBE) which insures employers, the self-employed, and their dependents. In 2003, these three schemes had more than 8.9 million beneficiaries, thus offering healthcare coverage to 85% of the Greek population [52]. The Statistical Departments of these three SHI funds (in response to multiple visits and written requests/questionnaires) provided us with annual accounting data covering the fiscal years 2001-2003, covering payments for hospital care, numbers of beneficiaries hospitalized, and numbers of days of hospitalization, all by contracted hospital provider ownership type. All data collected pertained to hospitals separated by ownership type and specialty-level aggregate information. No hospital- or patient-level information was available.

### Data analysis

Descriptive statistical analysis of relevant indicators (including hospital bed density by 1, 000 population, average hospital bed capacity, and average hospital occupancy rate), was performed using NSS data. Three additional indicators (nurse staffing density by hospital bed, average length of patient stay, and SHI payment per patient discharged), using both NSS and SHI data, were calculated, further analyzed, and used for comparative evaluation of PFP and public hospitals. Statistical comparisons between indicators (differences or ratios) were performed using the Confidence Interval Analysis (CIA) statistical package [55].

## Results

### PFP and public hospital characteristics

As shown in Table 1, PFP hospitals comprised 179 private clinics in 2003, representing 28.1% of the hospital beds of Greece. The majority of private clinics are of small- or medium-bed capacity (in 2003, 75.4% of PFP clinics had fewer than 100 beds per clinic) despite the fact that during the period examined many small units closed (77.5% of PFP clinics had fewer than 100 beds in 2001). In contrast, the number of larger private sector units (> 100 beds per clinic) remained steady. Most private clinics are general and psychiatric facilities, and the last showed a clear tendency to increase in number during the period examined (thus, between 2001 and 2003, PFP psychiatric hospital beds as a total of PFP beds increased by 2.2 percentage points).

**Table 1:** Hospital characteristics by ownership type

	2001		2002		2003	
	
	PUBLIC	PFP	PUBLIC	PFP	PUBLIC	PFP
**Hospitals, n**	139	191	141	179	142	179
Hospital beds, n (% total hospital beds in Greece)*	36, 186 (69.2)	15, 038 (28.8)	36, 142 (69.8)	14, 460 (27.9)	35, 814 (69.2)	14, 528 (28.1)
**Hospital beds per 1, 000 population**	3.30	1.37	3.29	1.32	3, 25	1, 32
**Average hospital bed capacity**	260	79	256	81	252	81
**Hospitals by hospital bed capacity, n (%)**						
1-40 beds	11 (7.9)	76 (39.8)	13 (9.2)	67 (37.4)	14 (9.9)	67 (37.4)
41-100 beds	24 (17.3)	72 (37.7)	26 (18.4)	69 (38.6)	26 (18.3)	68 (38.0)
100-500 beds	86 (61.9)	43 (22.5)	84 (59.6)	43 (24.0)	85 (59.9)	44 (24.6)
> 500 beds	18 (12.9)	0 (0.0)	18 (12.8)	0 (0.0)	17 (12.0)	0 (0.0)
**Hospital beds by hospital specialty, n (%)**						
General	26, 193 (72.4)	7, 492 (49.8)	26, 753 (74.0)	7, 343(50.8)	27, 114 (75.7)	7, 215 (49.7)
Mixed	0 (0.0)	1, 370 (9.1)	0 (0.0)	998 (6.9)	0 (0.0)	979 (6.7)
Maternity	384 (1.1)	1, 007 (6.7)	384 (1.1)	930 (6.4)	384 (1.1)	906 (6.2)
Psychiatric	5, 631 (15.6)	4, 386 (29.2)	5, 561 (15.4)	4, 392 (30.4)	4, 929 (13.8)	4, 567 (31.4)
Pediatric	1, 517 (4.2)	46 (0.3)	1, 529 (4.2)	77 (0.5)	1, 512 (4.2)	74 (0.5)
Other specialty†	2, 461 (6.8)	737 (4.9)	1, 915 (5.3)	720 (5.0)	1, 875 (5.2)	787 (5.4)
Patients discharged, n (% all patients discharged)*	1, 444, 743 (80.1)	327, 175 (18.2)	1, 499, 464 (79.8)	334, 760 (17.8)	1, 533, 030 (79.0)	356, 295 (18.4)
**Patients discharged by hospital specialty, n (%)**						
General	1, 271, 624 (88.0)	230, 468 (70.4)	1, 345, 272 (89.7)	249, 392 (74.5)	1, 380, 384 (90.0)	269, 992 (75.8)
Mixed	0 (0.0)	49, 136 (15.0)	0 (0.0)	35, 176 (10.5)	0 (0.0)	34, 312 (9.6)
Maternity	19, 376 (1.3)	27, 440 (8.4)	19, 896 (1.3)	28, 072 (8.4)	19, 880 (1.3)	28, 632 (8.0)
Psychiatric	12, 868 (0.9)	9, 691 (3.0)	14, 140 (0.9)	9, 720 (2.9)	14, 912 (1.0)	9, 935 (2.8)
Pediatric	72, 192 (5.0)	296 (0.1)	69, 376 (4.6)	432 (0.1)	66, 576 (4.3)	2, 280 (0.6)
Other specialty†	68, 683 (4.8)	10, 144 (3.1)	50, 780 (3.4)	11, 968 (3.6)	51, 278 (3.3)	11, 144 (3.1)
**Patients discharged by patient insurance scheme, n (%)**						
IKA	516, 056 (35.7)	125, 474 (38.4)	524, 836 (35.0)	154, 540 (46.2)	566, 719 (37.0)	168, 236 (47.2)
OGA††	543, 408 (37.6)	18, 458 (5.6)	483, 125 (32.2)	20, 952 (6.3)	203, 041 (13.2)	20, 719 (5.8)
TEBE	67, 594 (4.7)	10, 575 (3.2)	68, 446 (4.6)	13, 661 (4.1)	64, 931 (4.2)	16, 981 (4.8)
Other insurance scheme‡	317, 685 (22.0)	172, 668 (52.8)	423, 057 (28.2)	145, 607 (43.5)	698, 339 (45.6)	150, 359 (42.2)
**Average hospital (all specialties) occupancy rate**	81.8%	60.9%	82.4%	68.9%	101.4%	65.5%
**Average general hospital occupancy rate**	79.3%	50.7%	81.4%	60.7%	82.1%	61.0%

PFP: Private For-Profit. *All public, private for-profit and private not for-profit hospitals are included; military hospitals are not included.

† Other Specialty: Includes surgical clinics, eye clinics, oncology hospitals, dermatological hospitals, and infectious disease hospitals.

†† The decline in OGA beneficiaries discharged from public hospitals during 2002-2003 is a logistical consequence rather than an actual fact. Between March 2002 and December 2004 the Greek government erased the debt of OGA to public hospitals; this amounted to 941 million Euros.

‡ Other Insurance Schemes: includes patient beneficiaries of 32 Social Health Insurance Schemes, patients insured with Private Health Insurance Schemes, and the uninsured.

In 2003, the PFP hospital sector serviced more than 356, 000 patients, thus 18.4% of all hospital patients discharged (Table 1). During 2001-2003, PFP clinics showed an increase in productivity; the mean annual rise in the number of patients discharged was 4.4% per year. In addition, occupancy increased from 60.9% in 2001 to 65.5% in 2003. Three of every four patients discharged from the private sector were hospitalized in general PFP clinics, whereas single-specialty private clinics (maternity and psychiatric) absorbed a large proportion of all patients discharged from single-specialty hospitals; in 2003, for example, 59.0% and 40.0% of patients discharged from maternity and psychiatric hospitals, respectively, had been treated in the PFP sector. Finally, in 2003, 57.8% of all patients discharged from private clinics were beneficiaries of the 3 largest (of 35 in all) public insurance schemes (IKA, OGA, and TEBE); this had increased by 10.6 percentage points from 2001 to 2003.

Table 1 shows that there were 142 public hospitals in 2003, representing 69.2% of all hospital beds and absorbing 79.0% of all hospitalizations. Compared with PFP clinics, public hospitals are larger (with an average bed capacity of 252 beds per hospital in 2003), have higher occupancy rates (82.1% for general public hospitals in 2003), and show, during the period examined, a lower mean annual increase in the number of patients discharged (3.0% per year) compared with their private counterparts.

### Performance comparison of PFP and public hospitals

The NSS data (Table 2) showed that, in 2003, the number of nurses per hospital bed in PFP hospitals was 0.55, thus approximately one nurse for every two beds. The lowest numbers in the private hospital sector were observed in psychiatric and special surgery facilities (0.26 and 0.33 nurses per bed, respectively, in 2003). Depending on the year studied, 57.2-60.9% of nurses working in the private sector were poorly qualified (with 0-2 years of training).

**Table 2:** Nurse staffing levels by hospital ownership type

	2001				2002				2003			
	
	PUBLIC	PFP			PUBLIC	PFP			PUBLIC	PFP		
	
			RATIO PUBLIC/PFP	95% CI			RATIO PUBLIC/PFP	95% CI			RATIO PUBLIC/PFP	95% CI
**Nurses per hospital bed**, **by hospital specialty**												
General	1.08	0.63	1.71	1.66, 1.76	1.15	0.71	1.63	1.59, 1.68	1.16	0.74	1.57	1.52, 1.61
Mixed	-	0.48	-	-	-	0.55	-	-	-	0.59	-	-
Maternity	0.84	0.53	1.57	1.36, 1.80	0.95	0.58	1.63	1.42, 1.87	0.92	0.61	1.51	1.32, 1.73
Psychiatric	0.49	0.24	2.02	1.88, 2.17	0.51	0.26	1.97	1.84, 2.11	0.59	0.26	2.28	2.13, 2.44
Pediatric	0.96	0.46	2.11	1.37, 3.42	0.98	0.73	1.34	1.03, 1.79	0.98	0.73	1.35	1.03, 1.80
Other specialty*	0.95	0.33	2.90	2.54, 3.33	0.86	0.31	2.76	2.40, 3.18	0.88	0.33	2.67	2.30, 3.05
Total	0.97	0.48	2.01	1.96, 2.07	1.03	0.53	1.93	1.89, 1.98	1.06	0.55	1.93	1.88, 1.97
**Nurses per hospital bed**, **by nurse training level**												
RNs†	0.39	0.19	2.07	1.99, 2.16	0.42	0.23	1.86	1.79, 1.93	0.44	0.22	2.00	1.92, 2.07
LPNs††	0.39	0.17	2.28	2.19, 2.38	0.45	0.22	2.04	1.96, 2.12	0.44	0.24	1.88	1.81, 1.95
Aides‡	0.19	0.12	1.55	1.47, 1.63	0.16	0.08	1.87	1.76, 1.99	0.18	0.09	1.89	1.78, 2.01
Total	0.97	0.48	2.01	1.96, 2.07	1.03	0.53	1.93	1.89, 1.98	1.06	0.55	1.93	1.88, 1.97
									
			**DIFFERENCE PUBLIC - PFP**	**95% CI**			**DIFFERENCE PUBLIC - PFP**	**95% CI**			**DIFFERENCE PUBLIC - PFP**	**95% CI**
									
**Nurses by training level**, **% total nursing staff**												
RNs†	40.3	39.1	1.1	-0.1, 2.3	41.1	42.8	-1.6	-2.9, -0.4	41.5	40.1	1.4	0.2, 2.6
LPNs††	40.2	35.5	4.8	3.5, 6.0	43.5	41.3	2.2	1.0, 3.4	41.9	43.0	-1.1	-2.3, 0.1
Aides‡	19.5	25.4	-5.9	-7.0, -4.8	15.3	15.9	-0.5	-1.5, 0.3	16.6	16.9	-0.3	-1.2, 0.6
Total	100.0	100.0			100.0	100.0			100.0	100.0		

PFP: Private for-profit *Other specialty: Includes surgery clinics, eye clinics, oncology hospitals, dermatological hospitals, and infectious disease hospitals.

†RNs: registered nurses (3 or 4 years training) ††LPNs: licensed nurses (2 years training) ‡Aides: nonprofessional staff.

In the public hospital sector (Table 2), the number of nurses per hospital bed in 2003 was 1.06. In all years examined and over all hospital specialties, both all-nurse and highly qualified nurse staffing densities per hospital bed were higher than in PFP clinics. However, between 2001 and 2003, the public/PFP divergence in nurse staffing rates was reduced because the mean annual increase in nurse numbers was higher in PFP hospitals than in public facilities (from 2001-2003 the mean annual increase in nurse numbers in PFP hospitals was 4.6% per year; the corresponding figure for public hospitals was 3.6% per year). Finally, the largest public/PFP differences in nurse-per-bed figures were observed in specialty and psychiatric units; in 2003, for example, the numbers of nurses per public/PFP hospital bed were 2.67 and 2.28, respectively.

An analysis of ALoS in PFP and public hospitals is shown in Table 3. We used NSS data to this end; information is included on all patients hospitalized in PFP and public hospitals regardless of insurance status. We found: (a) a higher unweighted ALoS in general and specialized (mainly surgical) PFP hospitals compared with public facilities; and, (b) a lower unweighted ALoS for maternity, psychiatric, and pediatric PFP clinics compared with their public counterparts. Calculations using SHI data, which include only patients/beneficiaries of the three largest SHI schemes in the country, showed that, in all years studied and among all hospital specialties, unweighted ALoS was significantly higher in PFP clinics compared with public hospitals.

**Table 3:** Length of patient stay by hospital ownership type

	2001				2002				2003			
	
	PUBLIC	PFP			PUBLIC	PFP			PUBLIC	PFP		
	
			RATIO PFP/PUBLIC	95% CI			RATIO PFP/PUBLIC	95% CI			RATIO PFP/PUBLIC	95% CI
**Unweighted length of stay**, **by hospital specialty**†												
General	5.96	6.01	1.008	1.007, 1.010	5.91	6.52	1.105	1.103, 1.107	5.89	5.95	1.011	1.009, 1.013
Mixed	-	4.80	-	-	-	9.22	-	-	-	4.01	-	-
Maternity	4.60	4.56	0.991	0.982, 0.999	4.90	4.40	0.898	0.891, 0.906	4.74	4.13	0.871	0.863, 0.878
Psychiatric	160.51	131.04	0.816	0.815, 0.818	133.41	131.17	0.983	0.981, 0.985	282.09	139.10	0.493	0.492, 0.494
Pediatric	4.66	5.59	1.200	1.143, 1.259	5.14	2.81	0.548	0.518, 0.580	4.97	3.37	0.678	0.662, 0.693
Other specialty*	10.66	31.84	2.988	2.976, 3.001	11.52	23.75	2.061	2.052, 2.071	9.66	19.78	2.048	2.038, 2.059
Total	7.48	10.21	1.366	1.364, 1.367	7.25	10.86	1.498	1.496, 1.500	8.65	9.75	1.127	1.126, 1.129
**Unweighted length of stay**, **by patient insurance scheme** **and by hospital specialty**††												
**IKA**												
General	5.68	9.09	1.601	1.597, 1.605	5.05	8.23	1.630	1.626, 1.633	4.89	8.84	1.810	1.806, 1.814
Psychiatric	36.52	47.45	1.299	1.293, 1.305	40.22	80.13	1.992	1.984, 2.001	41.62	60.62	1.457	1.450, 1.463
All specialties‡	6.10	14.59	2.392	2.388, 2.396	5.46	15.48	2.833	2.828, 2.838	5.27	15.30	2.903	2.899, 2.908
**OGA**												
Acute hospitals	4.19	7.37	1.760	1.749, 1.770	4.01	7.25	1.808	1.798, 1.818	4.30	6.71	1.561	1.552, 1.571
Long-term care hospitals	28.54	139.41	4.885	4.868, 4.901	23.66	138.78	5.865	5.845, 5.884	22.51	131.07	5.822	5.803, 5.841
All specialties	6.96	29.29	4.206	4.194, 4.218	6.26	28.39	4.533	4.520, 4.545	9.56	29.26	3.062	3.053, 3.070
**TEBE**												
All specialties	4.08	6.80	1.668	1.654, 1.682	3.98	4.59	1.151	1.142, 1.162	3.82	4.27	1.116	1.106, 1.125
**Total (IKA, OGA, TEBE)**	6.39	15.81	2.473	2.469, 2.476	5.73	16.12	2.815	2.811, 2.819	6.20	15.80	2.548	2.544, 2.551

PFP: Private for-profit, Length of Stay: Days, *Other specialty: Includes surgery clinics, eye clinics, oncology hospitals, dermatology hospitals, and infectious disease hospitals.

†LoS refers to all patients discharged from public and PFP hospitals, regardless of insurance status, according to NSS data.

††LoS refers only to patients discharged from public and PFP hospitals who were beneficiaries of the three SHI schemes included in our sample, according to SHI data.

‡ Distinctions between hospitals by specialty differ between the SHI and NSS datasets.

Finally, Table 4 summarizes our results on SHI payments by hospital ownership type. The SHI data showed that, from 2001 to 2003, the three largest insurance schemes paid more than 765 million Euros to contracted private clinics. Depending on the year studied, payments to private clinics represented 18.7-29.1% of total hospital expenditure by SHIs. For OGA and TEBE, for which the relevant disaggregated information was available (data not presented in Table 4), fixed per diem fees in 2003 represented 48.4% and 17.0%, respectively, of total payments to private clinics; the rest constituted fee-for-service payments for procedures and examinations performed during hospitalization. In contrast, fixed per diem fees represented larger proportions of total payments by OGA and TEBE to public hospitals (57.4% and 48.7%, respectively, in 2003).

**Table 4:** Social Health Insurance fund payments for care by hospital ownership type

	2001				2002				2003			
	
	PUBLIC	PFP	TOTAL		PUBLIC	PFP	TOTAL		PUBLIC	PFP	TOTAL	
**SHI payments for hospital care, millions of € (%)**	897.8 (81.3)	206.9 (18.7)	1104.6 (100.0)		832.7 (76.8)	251.4 (23.2)	1084.2 (100.0)		747.6 (70.9)	307.0 (29.1)	1054.6 (100.0)	
									
			**RATIO PFP/PUBLIC**	**95% CI**			**RATIO PFP/PUBLIC**	**95% CI**			**RATIO PFP/PUBLIC**	**95% CI**
									
**SHI payments for hospital care** **per patient discharged, €**												
IKA	877.1	1298.5	1.481	1.480, 1.481	783.8	1234.7	1.575	1.574, 1.576	919.7	1447.7	1.574	1.573, 1.575
OGA	743.6	1786.9	2.403	2.400, 2.405	786.9	2169.2	2.757	2.754, 2.759	900.9	2101.3	2.333	2.330, 2.335
TEBE	607.3	1036.9	1.707	1.706, 1.708	602.0	1109.6	1.843	1.842, 1.844	669.2	1170.5	1.749	1.748, 1.750
Total (IKA, OGA, TEBE)	796.6	1338.9	1.681	1.680, 1.682	773.6	1329.2	1.718	1.717, 1.719	895.6	1490.6	1.664	1.663, 1.665

PFP: Private for-profit

SHI: Social Health Insurance

As shown in Table 4, in all years examined, over all hospital specialties and for all insurance schemes included in our study sample, SHI payments (including per diem fee, plus additional fee-for service payments for services provided during hospitalization) for hospital care per discharge were higher in PFP clinics than in public hospitals.

## Discussion

According to the results of our study significant performance differences exist between PFP and public hospital-care providers operating within the mixed healthcare system in Greece. PFP hospitals have lower bed capacity, lower occupancy rates and lower nurse (total and high-qualified) staffing rates compared to public hospitals and are associated with higher unweighted length of stay and higher payments per discharge, at least in the case of discharged patients that are beneficiaries of the Greek SHI funds.

Most of our findings are in line with empirical evidence from other developed countries, but are here highlighted for the first time in the case of Greece. The data have long been neglected by health policy analysts and decision makers. The data collected and analyzed came from a wide range of official sources, organizations, and departments within organizations, and covered all hospital providers in Greece, with the exception of the few private nonprofit (charitable) providers. Additionally, the datasets used in our study have remained unexplored for years because the existence of these data were unknown to government health policy agencies and, in some instances, even to the central administrative bodies of relevant organizations.

Our findings show that the private sector plays a key role in the provision of hospital services in the country. The majority of such institutes remain small- and medium-bed capacity facilities, with low occupancy rates, highly dependent on public SHI funds. Earlier studies had shown that in Greece the probability of treatment in a private hospital is positively related to high family income and to the existence of private health insurance coverage [56]. Our study confirms these findings since the beneficiaries of the three largest public sickness funds - mainly coming from low and middle income classes - that correspond to 85% of the Greek insured population, represent only 6 out of 10 patients treated in private clinics.

The PFP hospital market has undergone several structural changes over the past two decades. In the interval since 1996, increased competition has forced many small and single-specialty clinics into bankruptcy and closure. Others have formed powerful companies (via mergers and acquisitions) owning several small facilities as well as a few luxurious units of high-bed capacity and high occupancy rates; these facilities cater for (principally) private patients and beneficiaries of private healthcare insurance [57]. Such developments within the private healthcare market have increased the heterogeneity of private hospital providers, a fact only partly reflected in the findings of our current research.

The performance of PFPs and public hospitals was investigated using measures referring to structural/input (bed capacity, occupancy rate, nurse staffing) and efficiency (unweighted ALoS, SHI payment per discharge) differences between hospital providers by ownership type. Although performance assessment requires a multi-dimensional approach [58,59], such as that used in the present study, it has been asserted that outcome indicators (such as mortality rates, health outcomes, and patient satisfaction) are stronger quality measures than are those of input and process [60]. The lack of relevant data meant that we could not include these useful measures in our analysis.

Despite the limitations mentioned above, our present research reveals significant differences in crucial performance aspects of PFPs and public hospitals in Greece.

PFP hospital service providers use fewer, and more poorly qualified, nurses than do public hospitals, regardless of hospital specialty or year (2001-2003). This finding is in agreement with international empirical evidence showing that PFP providers (including general and psychiatric hospitals, dialysis centers, and nursing homes) have lower total nurse [61-63] and registered nurse [62,64,65] staffing levels than do private nonprofit competitors. In Greece, evidence on nurse staffing level by hospital ownership type became available for the first time in 1995 [66], and since then the relevant data were thought to be non-existent [67]. Several health policy analysts have sought to explain the 1993 PFP/public nurse staffing difference as reflecting better staff management practices in the private sector. The implication was that PFP hospitals in Greece used their smaller nursing staff more efficiently than did public hospitals, without compromising quality of care [68,69]. International evidence shows that the need to maximize profits forces PFP providers to minimize expenditure on nursing, and especially on highly qualified nurses, a practice related in many instances to poor healthcare outcomes [27,31,64]. Understaffing of PFP clinics owing to diversion of funds from clinical needs to profit is the most obvious explanation of the surprisingly low nurse staffing rates in PFP hospitals found in our present study.

We also found that the unweighted ALoS values in most cases were higher in PFP clinics than in public hospitals (regardless of hospital specialty or year of study according to SHI data, only in general and specialized hospitals according to NSS data). We accept, of course, that ALoS is highly dependent on illness severity, and the lack of case mix adjustment (because the relevant data were not available) constitutes a serious limitation of our study. Nevertheless, based on the consensus among health policy researchers that the most severely ill patients in Greece are treated in the public sector [47,67,69], one would have expected the result to be the opposite of what was observed.

Additionally, we consistently found that ALoS of PFP clinics was significantly higher when calculated using SHI data compared to calculations using NSS data. This divergence in ALoS of private hospitals between the two data sources probably reflects the fact that SHI dataset includes only discharged patients whose public insurance fund compensate the hospital on a per diem basis, excluding at the same time private patients or private insured patients who compensate the hospital on a fixed per discharge basis. International empirical evidence has shown that hospital reimbursement on a per diem basis creates incentives for private profit maximizing hospitals to increase length of patient stay, longer than medically required [37]. This observation of our study raises ethical concerns whether supplier-induced demand plays a role in the Greek private hospital sector and needs further investigation by future research.

Finally, we found that SHI payment-per-discharge was significantly higher in PFP clinics compared with public hospitals, regardless of the year (2001-2003) of study or the SHI scheme evaluated. This is in agreement with international empirical evidence; payments for care are higher in PFP hospitals than in private nonprofit institutions [30]. USAs Medicare is a public insurance fund that operates as do the Greek SHI schemes evaluated in the current study. The evidence shows that higher payments to PFP hospital providers are attributable to malpractice including upcoding of diagnosis-related groups [15], formal and informal relationships with outpatient suppliers of healthcare services such as skilled nursing facilities and home healthcare [16,70], and incentives that depend on the method of reimbursement associated with an increase in the average length of stay (as has been shown in contracted rehabilitation hospitals) [71]. In the case of the Greek SHI funds studied here, the most likely explanation of the higher payment per discharge to PFP hospital providers seems to be either an increase in the ALoS charged by private clinics or the excessive use of diagnostic services, the reimbursement of which is not included in the fixed per diem fee.

Our results indicate that if the three SHI schemes included in our sample had directed all of their hospitalized beneficiaries to public rather than PFP hospitals, they could have saved 122.2 million Euros in 2003 and more than 304 million Euros during the 3-year period examined in the present study. This finding is of particular interest especially in the context of the current economic crisis that manifested in Greece in the form of public-debt crisis; since it implies that public administration's incompetence to formulate a comprehensive and mandatory system of data submission caused a constant money flow from public funds to private providers, that contributed to the dramatic deterioration in Greece's public accounts.

## Conclusions

In a mixed European healthcare system (such as that of Greece), significant performance differences are evident between PFP and public hospital service providers, in terms of several indicators such as average bed capacity, average occupancy rate, nurse staffing rates, average length of patient stay, and SHI payment per discharge. The public hospital sector performed better than did PFP competitors in terms of all measures included in our study. Our findings do not mean that the performance or quality of care at most public facilities in Greece is excellent or even adequate. Rather, most of our findings, in agreement with international empirical evidence, raise the concern that for-profit ownership of hospital facilities negatively affects the quality and cost of patient care.

Close monitoring and continuous comparative assessment of healthcare providers by ownership type, a research and policy practice neglected in most European countries and almost unknown to date in Greece, can offer useful data facilitating rational health policy decisions on the appropriate balance of, or even the need for, a public/private hospital mix.

## Competing interests

The authors declare that they have no competing interests.

## Authors' contributions

EK conducted the research, collected and analyzed the data, and prepared the manuscript. MG contributed to data analysis and manuscript editing. SG and ES contributed to data analysis and the final editing of the manuscript. ND contributed to the study design. AB supervised and co-ordinated the study design, data analysis, and interpretation, as well as preparation of the manuscript. All authors have read and approved the final manuscript.

## Pre-publication history

The pre-publication history for this paper can be accessed here:

http://www.biomedcentral.com/1472-6963/11/234/prepub
